# Responsible artificial intelligence in medical imaging: a systematic review

**DOI:** 10.3389/fdgth.2026.1884692

**Published:** 2026-07-16

**Authors:** Nafiz Fahad, Ridwan Jamal Sadib, Rakib Hossain Sajib, Md Kishor Morol, Dip Nandi, Tze Hui Liew

**Affiliations:** 1 ELITE Research Lab, New York, NY, United States; 2 Faculty of Information Science and Technology, Multimedia University, Melaka, Malaysia; 3 Faculty of Science and Technology, American International University-Bangladesh, Dhaka, Bangladesh; 4 Department of Computer Science and Engineering, Begum Rokeya University, Rangpur, Bangladesh; 5 Department of Computer Science, American International University-Bangladesh, Dhaka, Bangladesh; 6 Centre for Intelligent Cloud Computing (CICC), COE of Advanced Cloud, Faculty of Information Science & Technology, Multimedia University, Melaka, Malaysia

**Keywords:** clinical trust, deep learning, disease detection, explainable AI, fairness, healthcare AI, medical imaging, privacy

## Abstract

**Introduction:**

Responsible artificial intelligence (AI) in medical imaging requires more than high diagnostic accuracy; it also requires transparent reasoning, equitable performance across patient subgroups, privacy protection, calibrated uncertainty, and clinical trustworthiness.

**Methods:**

This PRISMA-informed systematic review synthesized 24 studies published between 2020 and 2025 that used AI or deep learning for disease detection or diagnostic support in X-ray, CT, MRI, mammography, ultrasound, dermoscopy, retinal fundus imaging, optical coherence tomography, and abdominal CT. PubMed, Scopus, Web of Science, IEEE Xplore, ScienceDirect, SpringerLink, and Google Scholar were searched, and extracted evidence was appraised qualitatively using adapted QUADAS-2 and PROBAST-AI domains.

**Results:**

The included studies covered lung diseases, COVID-19, pneumonia, lung cancer, breast cancer, melanoma and other dermatological disorders, brain tumors, diabetic retinopathy, chest abnormalities, and pancreatic ductal adenocarcinoma. Explainability methods such as Grad-CAM, Grad-CAM++, LIME, SHAP, saliency maps, and layer-wise relevance propagation dominated the evidence base, whereas fairness, privacy-preserving learning, uncertainty estimation, and human-centered clinical trust were represented by fewer studies. Several papers reported accuracy or sensitivity above 90%, but these values should be interpreted cautiously because many studies relied on internal validation, curated public datasets, class-balanced splits, augmentation, or limited demographic reporting.

**Discussion:**

Responsible medical-imaging AI should be evaluated through multidimensional evidence, including external and subgroup validation, calibration, privacy risk analysis, clinician-centered explanation assessment, workflow integration, regulatory readiness, and post-deployment monitoring.

## 1 Introduction

Artificial intelligence can support disease detection from radiographic and clinical imaging data, including CT scans, MRI scans, mammograms, ultrasound images, dermoscopic images, retinal fundus images, and optical coherence tomography. Recent models have been applied to lung disease, cancer, dermatology, neurology, ophthalmology, and breast imaging. However, clinical deployment requires more than high classification accuracy. Responsible AI in medical imaging must consider generalizability, privacy, fairness, explainability, uncertainty, safety, and physician trust.

The revised manuscript explicitly clarifies that explainability is the most common responsible-AI component in the included studies, but it is not equivalent to responsible AI by itself. Heatmaps and feature-attribution methods can help visualize what image regions influenced a prediction, yet these methods do not automatically prove clinical validity, causal reasoning, or safety. Therefore, this review separates the evidence for explainability, fairness, privacy-preserving learning, uncertainty, robustness, and clinical trust instead of treating them as interchangeable concepts.

In lung disease studies, explainability has been used to highlight potentially relevant regions in chest X-ray and CT images during diagnosis of lung cancer, COVID-19, pneumonia, and tuberculosis (5, 6, 23, 24). Breast cancer, skin lesion, and brain tumor studies have similarly used visual explanations to improve interpretability of CNN, transformer, ensemble, and segmentation-classification models (13, 14, 16, 17).

Fairness and privacy represent additional dimensions of responsible AI. Bias can arise when algorithms perform differently across age, sex, race, skin tone, disease severity, scanner type, hospital site, or geographic population. For example, Benčević et al. (8) reported lower skin lesion segmentation performance for darker skin tones, Lin et al. (2) used supervised contrastive learning to reduce demographic bias in chest radiograph diagnosis, and Tayebi Arasteh et al. (1) evaluated differential privacy together with diagnostic performance and fairness. Privacy-preserving approaches, including federated learning and differential privacy, are particularly important because clinical imaging data are sensitive and are often restricted from centralized sharing (1, 12).

The revision also adds selected methodological references from major AI and biomedical informatics venues suggested by the reviewers. These works broaden the discussion of data completeness, data bias, generative-model reliability, and feature-selection robustness: generative AI-based data completeness augmentation for data-driven smart healthcare, generative model perception rectification for diversity-quality trade-off, self-supervised standardization for mitigating healthcare data bias, and a deep neural network optimization framework using optimal transport bridge feature selection and sparse representation (25–28). These references are not counted among the original 24 disease-detection imaging studies unless they met the eligibility criteria, but they strengthen the discussion of responsible data development and bias mitigation.

**Figure 1:**
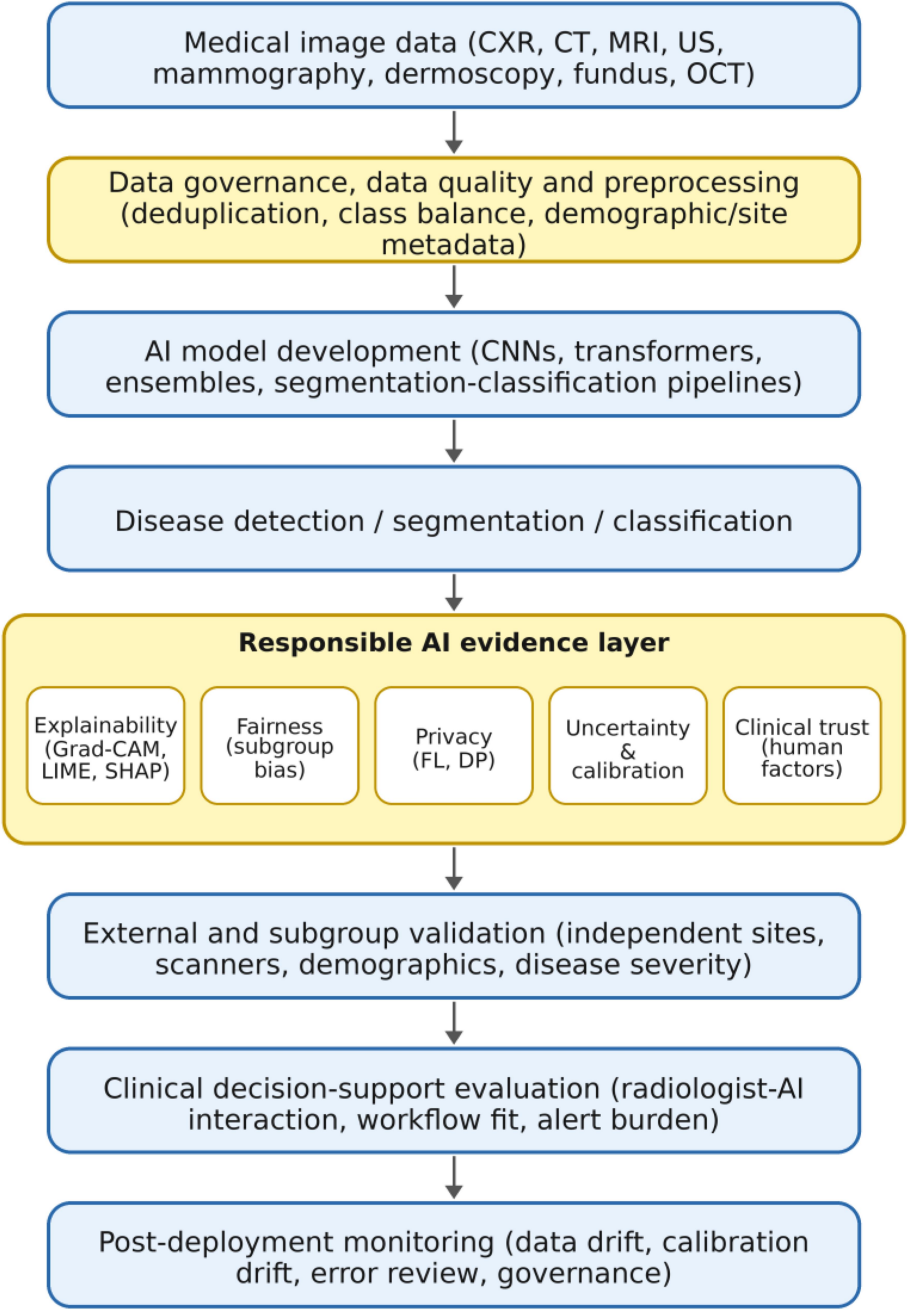
Responsible AI framework for medical imaging disease detection.

The Figure 1 highlights the integration of data governance, explainability, fairness, privacy, uncertainty, calibration, clinician-centered trust, external validation, clinical decision-support evaluation, and post-deployment monitoring. Yellow boxes indicate newly emphasized reviewer-requested elements.

Moreover, the remainder of this article is organized as follows: Section 2 describes the PRISMA-informed scoping mini-review approach, including the search strategy, eligibility criteria, data extraction, and methodological appraisal. Sections 3–6 synthesize the performance, responsible-AI evidence domains, quality and bias concerns, and clinical translation recommendations, while Sections 7 and 8 present the limitations, future research directions, and conclusion.

## 2 Methods: PRISMA-informed scoping mini-review approach

This article is clarified as a PRISMA-informed scoping mini review rather than a full systematic review or meta-analysis. The objective was to map responsible-AI evidence in medical imaging disease detection, identify methodological gaps, and synthesize clinical implications across heterogeneous modalities, diseases, and AI architectures. Because the included studies differed substantially in disease area, imaging modality, dataset size, model architecture, validation type, and performance metrics, no quantitative meta-analysis was conducted.

Information sources and date range. PubMed, Scopus, Web of Science, IEEE Xplore, ScienceDirect, SpringerLink, and Google Scholar were searched for studies published between 2020 and 2025. Citation chaining and targeted searching of relevant biomedical AI venues were also used to identify additional studies. The exact database strings are provided in Supplementary Table S1 so that the search can be reproduced and updated.

Duplicate management and screening. Records were exported to a reference manager and screened for duplicates using DOI matching, title matching, and manual verification of author-year combinations. Duplicate records were removed before title and abstract screening. Titles and abstracts were screened against the eligibility criteria, followed by full-text assessment. Disagreements or ambiguous cases were resolved through consensus among the authors.

Eligibility criteria: Studies were included if they: (i) used medical imaging data, (ii) applied AI/deep learning for disease detection, classification, segmentation, or diagnostic decision support, (iii) reported diagnostic or model performance information, and (iv) included at least one responsible-AI element such as explainability, fairness, privacy, uncertainty, robustness, external validation, or human-centered clinical evaluation. Studies were excluded if they were unrelated to medical imaging, did not address disease detection or diagnostic support, lacked a responsible-AI component, or did not provide extractable information for synthesis.

Data extraction: Extracted information included imaging modality, disease area, dataset source, model family, responsible-AI component, explainability method, fairness subgroup, privacy-preserving method, uncertainty/calibration method, validation type, reported performance, external validation status, limitations, and clinical translation relevance. The extracted study characteristics, responsible-AI evidence domains, methodological quality appraisal, and clinical translation recommendations are summarized in Tables 1–5.

**Table 1:** Performance of responsible AI in medical imaging with validation context and limitations.

Study	Modality/dataset	Responsible-AI component	Disease area	AI method	Reported performance	Validation type/limitations
Hasan et al. (5)	Chest CT and X-ray; COVID-19 radiography and CT datasets	Grad-CAM; DCGAN balancing	Lung abnormalities	CTXNET/compact convolutional transformer	99.77% CT accuracy; 95.37% X-ray accuracy	Internal/benchmark validation; no prospective external hospital validation; possible augmentation and curation effects
Rajpoot et al. (4)	COVIDx CXR-3 and SARS-CoV-2 CT	LIME; SHAP; Grad-CAM; Grad-CAM++	COVID-19	Ensemble CNN: DenseNet169, ResNet50, VGG16	99.00% X-ray accuracy; 96.18% CT accuracy	Benchmark validation; modality-specific datasets; external clinical workflow not tested
Mahamud et al. (6)	Chest X-ray lung disease dataset	SHAP; LIME; Grad-CAM; Grad-CAM++	COVID-19; pneumonia; tuberculosis; normal	Enhanced DenseNet201 transfer learning	99.20% accuracy	Internal testing; limited demographic/site reporting; external generalizability uncertain
Hammad et al. (23)	Chest CT-scan image dataset	Grad-CAM	Lung cancer subtypes	Custom CNN	93.06% accuracy; 97.25% AUC	Internal dataset evaluation; scanner/site heterogeneity and clinician validation not clear
Fatima et al. (17)	MIAS mammography dataset	Grad-CAM	Breast cancer classification	ResNet50, VGG16, VGG19, GoogleNet	99.24% accuracy using ResNet50	Small/curated public dataset; stratified CV helps internal reliability but not external translation
Zou & Miao (16)	Breast ultrasound; 8,116 images	Grad-CAM++	Benign vs. malignant breast cancer	Hybrid VGG16 + DenseNet121 + Xception	97.14% accuracy	Internal evaluation; site/scanner/demographic subgroup reporting limited
Kakon et al. (13)	BR35H brain MRI dataset	Grad-CAM++; LIME; SHAP	Brain tumor detection	EfficientNetB7, InceptionV3, Xception, LightGBM meta-learner	99.83% accuracy	High internal performance; risk of public-dataset bias and limited prospective validation
Mastoi et al. (12)	Kaggle Brain Tumor MRI; 7,023 images	Federated learning; Grad-CAM; saliency maps	Brain tumor classification	Federated GoogLeNet	94.24% final-round accuracy	Federated setting appears simulated/benchmark-based; real inter-hospital governance and leakage risk need testing
Akram et al. (9)	APTOS 2019 and DDR retinal fundus datasets	Bayesian uncertainty estimation	Diabetic retinopathy	DenseNet-121 with MC Dropout/MFVI	97.68% accuracy using BCNN-MC Dropout	Uncertainty useful, but calibration, referral thresholds, and external clinical workflow need further study
Benčević et al. (8)	PH2, Waterloo, Dermofit, ISIC 2018	Skin-tone bias evaluation	Skin lesion segmentation	U-Net with ResNet-18 encoder	DSC: 0.904 light; 0.878 medium; 0.744 dark skin	Strong fairness signal; evaluates disparity but does not fully solve mitigation or clinical deployment
Lin et al. (2)	MIDRC and NIH Chest X-ray14	Fairness via supervised contrastive learning	COVID-19 and thoracic abnormalities	DenseNet-121 + supervised contrastive learning	Reduced Delta mAUC bias across sex, race, and age	Fairness mitigation evaluated; prospective and workflow validation still needed
Tayebi Arasteh et al. (1)	UKA-CXR and PDAC CT datasets	Differential privacy and fairness analysis	Chest disease and pancreatic cancer	ResNet9 with DP-SGD	UKA-CXR AUROC 87.36% at epsilon = 7.89; PDAC AUROC 99.28% at epsilon = 8	Demonstrates privacy-utility-fairness trade-off; subgroup-specific deterioration must be monitored

**Table 2:** Evidence distribution across responsible-AI domains.

Responsible-AI domain	Evidence coverage	Examples	Modalities/diseases	Main interpretation
Explainability	Most included studies	Grad-CAM, Grad-CAM++, LIME, SHAP, saliency maps, RELPROP	Lung, COVID-19, breast, brain, dermatology, retinal/OCT	Useful for transparency but does not automatically create trust; requires expert validation and clinical task alignment
Fairness and bias	3 core included studies plus added methodological references	Skin-tone analysis; sex/race/age Delta mAUC; differential privacy fairness analysis; self-supervised standardization	Dermatology, chest radiography, large-scale medical imaging	Few studies measured or mitigated subgroup bias; subgroup definitions and reporting are inconsistent
Privacy-preserving AI	2 core included studies	Federated learning; differential privacy/DP-SGD	Brain tumor MRI; chest disease; PDAC CT	Promising for multi-center learning but practical governance, communication burden, model leakage, and utility trade-offs remain under-tested
Uncertainty and calibration	1–2 included studies	Bayesian CNN, MC Dropout, variational inference	Diabetic retinopathy; brain tumor imaging	Uncertainty can triage low-confidence cases, but calibration metrics and action thresholds are rarely reported
Clinical trust and usability	1 human-centered XAI study	Clinician-centered XAI evaluation	Chest radiology	Trust requires workflow fit, human factors, alert fatigue evaluation, responsibility allocation, and patient-outcome evidence
Robustness/external validation	Limited and heterogeneous	Multi-dataset testing, cross-dataset evaluation, subgroup testing	Skin lesions, chest imaging, brain tumor and other modalities	External validation and performance drop reporting are inconsistent; no included study establishes broad deployment readiness

**Table 3:** Adapted QUADAS-2/PROBAST-AI methodological appraisal of included studies.

Study	Dataset/participants concern	Model/analysis concern	Fairness/privacy concern	Clinical applicability concern	Overall concern	Reason for rating
Hasan et al. (5)	Moderate	Moderate	Unclear	High	High	High accuracy but external hospital validation, subgroup analysis, and prospective use not demonstrated
Rajpoot et al. (4)	Moderate	Moderate	Unclear	High	High	Multiple XAI methods; benchmark validation not enough for clinical translation
Mahamud et al. (6)	Moderate	Moderate	Unclear	High	High	Demographic/site variation and external validation not clearly assessed
Hammad et al. (23)	Moderate	Moderate	Unclear	High	High	CT lung cancer model needs scanner/site diversity and clinician validation
Fatima et al. (17)	High	Moderate	Unclear	High	High	MIAS is small/curated; stratified CV does not substitute for external testing
Zou & Miao (16)	Moderate	Moderate	Unclear	High	High	Large ultrasound dataset but limited subgroup and workflow evidence
Kakon et al. (13)	High	Moderate	Unclear	High	High	Very high reported accuracy; public-dataset and leakage/generalization concerns need checking
Mastoi et al. (12)	Moderate	Moderate	Unclear	High	High	Federated learning relevant, but real multi-hospital validation and leakage analysis needed
Akram et al. (9)	Moderate	Moderate	Unclear	Moderate	Moderate	Uncertainty considered; calibration and referral decision thresholds still needed
Benčević et al. (8)	Low/Moderate	Low	Low	Moderate	Moderate	Strong subgroup bias evaluation; mitigation and prospective clinical impact still limited
Lin et al. (2)	Low/Moderate	Low	Low	Moderate	Moderate	Fairness mitigation across sex/race/age; prospective clinical validation not established
Tayebi Arasteh et al. (1)	Low/Moderate	Low/Moderate	Low	Moderate	Moderate	Strong privacy-fairness analysis; privacy-utility subgroup effects require deployment monitoring
Nazir et al. (7)	Moderate	Moderate	Unclear	High	High	External generalizability concerns noted; exact drop not harmonized across metrics
Lakshmi et al. (10)	Moderate	Moderate	Unclear	High	High	Segmentation-classification pipeline; external validation and clinical assessment limited
Iftikhar et al. (11)	Moderate	Moderate	Unclear	High	High	XAI feature identification useful; clinical explanation validation unclear
Singh et al. (14)	Moderate	Moderate	Unclear	High	High	Cross-dataset performance promising but deployment-ready evidence still limited
Saharan et al. (15)	Moderate/High	Moderate	Unclear	High	High	Breast cancer XAI; external site, calibration, and subgroup reporting needed
Acosta-Jiménez et al. (18)	Moderate	Moderate	Unclear	High	High	Mammography XAI; clinical reader study and external validation needed
Yoshida et al. (19)	Moderate	Moderate	Unclear	Moderate/High	Moderate/High	OCT exploration clinically relevant; generalizability and patient-outcome impact unclear
Fiaz et al. (20)	Moderate	Moderate	Unclear	High	High	Skin lesion segmentation-classification; fairness by skin tone not fully established
Mahmud et al. (21)	Moderate	Moderate	Unclear	High	High	Melanoma detection with XAI; subgroup and prospective validation needed
Badhon et al. (22)	Moderate/High	Moderate	Unclear	High	High	Skin disease classification with Grad-CAM; limited clinical trust and fairness evidence
Ihongbe et al. (3)	Moderate	Low/Moderate	Not applicable	Moderate	Moderate	Human-centered XAI evaluation strengthens clinical trust discussion
Veeramani et al. (24)	Moderate/High	Moderate	Unclear	High	High	Lung disease XAI; external clinical validation and subgroup testing needed

**Table 4:** Common limitations and recommended reporting improvements for included studies.

Common limitation	Why it matters	Recommended reporting/mitigation
Public or curated datasets	Many studies used public repositories or curated challenge datasets that may not capture real clinical heterogeneity.	Report data provenance, scanner/site diversity, disease severity, and demographic distribution.
Internal validation dominance	Many high accuracies were based on internal splits or cross-validation only.	Add independent external test sets and report performance drop from internal to external validation.
Small or imbalanced samples	Some disease classes or subgroups were small or under-represented.	Use subgroup-aware sampling, transparent class counts, and confidence intervals.
Potential leakage/augmentation artifacts	Patch-level splitting, duplicated images, or synthetic augmentation may inflate performance if not controlled.	Use patient-level splitting, deduplication, and leakage checks.
Limited demographic fairness reporting	Age, sex, race, skin tone, hospital site, and scanner type were often not reported.	Report subgroup performance and mitigation strategies.
Limited calibration and uncertainty reporting	Accuracy/AUC were prioritized over calibration, Brier score, ECE, or uncertainty thresholds.	Report calibration curves, expected calibration error, and decision thresholds for uncertain cases.
Limited clinician involvement	Most XAI heatmaps were not evaluated by radiologists or domain experts.	Include reader studies, usability testing, and explanation-actionability assessment.
No prospective evaluation	Retrospective studies dominated the evidence.	Use prospective silent trials, workflow studies, and patient-outcome evaluation before deployment.
Privacy and governance gaps	Federated learning and DP were discussed in few studies; governance and leakage risks were rarely tested.	Report privacy budget, attack analysis, governance model, and communication burden.
Post-deployment monitoring missing	Data drift, model drift, and long-term safety monitoring were not well covered.	Implement monitoring plans, periodic recalibration, and error review committees.

**Table 5:** External validation and performance-drop/disparity evidence.

Study/group	External or subgroup validation evidence	Performance drop/disparity reported	Clinical translation interpretation
Benčević et al. (8)	Multiple skin lesion datasets and skin-tone subgroup analysis	Light vs. dark skin DSC difference: 0.904 vs. 0.744; absolute drop 0.160	Important fairness warning; subgroup disparities must be reported and mitigated before clinical use
Lin et al. (2)	Multi-dataset chest radiograph fairness analysis with sex, race, and age	Accuracy drop not primary metric; reduced Delta mAUC bias reported	Fairness-aware learning can reduce disparity, but prospective workflow validation is still needed
Tayebi Arasteh et al. (1)	Large-scale privacy/fairness evaluation across chest disease and PDAC CT data	Performance changed with privacy budget; exact pooled drop not comparable across tasks	Privacy settings should be selected with utility, calibration, and subgroup fairness reporting
Nazir et al. (7)	External generalizability discussed for brain tumor prediction	Lower external generalizability noted; exact drop not harmonized in this mini-review extraction	External testing should be mandatory before clinical claims
Singh et al. (14)	Across-dataset brain tumor classification framework	Reported cross-dataset robustness, but performance-drop reporting is not standardized here	Cross-dataset testing is valuable but needs transparent site-level and patient-level reporting
Other high-accuracy XAI studies	Mostly internal validation or public benchmark testing	No consistent external drop reported	High accuracy should be interpreted as preliminary unless validated externally and prospectively

Methodological appraisal: A structured quality appraisal was added using adapted QUADAS-2 and PROBAST-AI domains. Because the included studies were heterogeneous and many were not diagnostic test accuracy studies in the strict clinical-trial sense, the appraisal was qualitative. The domains were: participants/dataset representativeness, index test/model transparency, reference standard/label quality, analysis and validation, fairness/privacy/clinical applicability, and overall concern. Ratings were categorized as low, moderate, high, or unclear concern.

**Figure 2:**
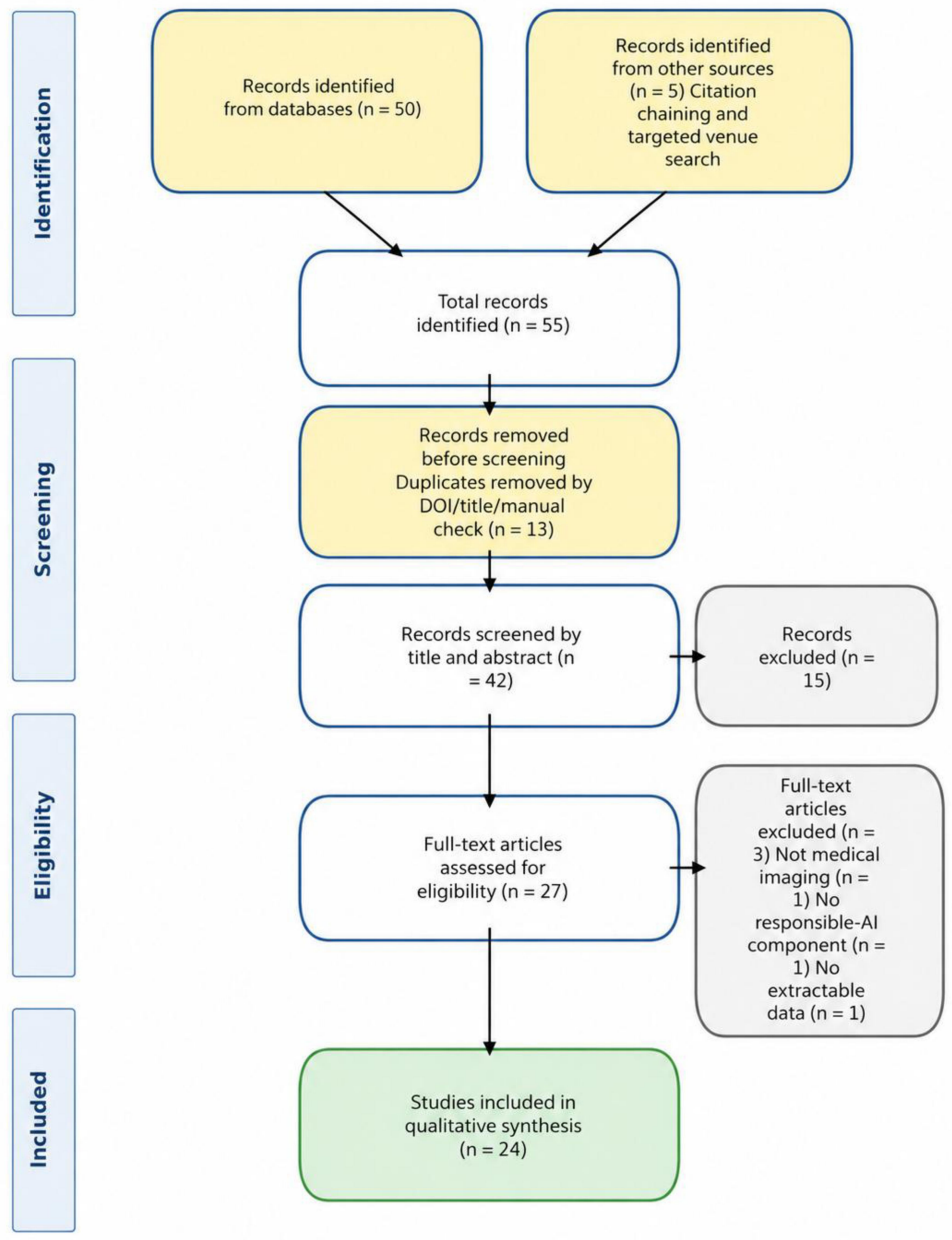
PRISMA 2020 flow diagram of study selection.

The Figure 2 includes database-specific identification counts, duplicate removal, title/abstract screening, full-text eligibility, reasons for full-text exclusion, and the final 24 included studies. The database counts preserve the original manuscript total and should be cross-checked against the authors’ saved search log before resubmission.

## 3 Performance of responsible AI in healthcare: balanced interpretation

The reviewed literature indicates that responsible-AI systems can achieve strong diagnostic performance across multiple medical imaging modalities. However, high accuracy alone is not sufficient evidence for clinical readiness. In this revised version, performance values are interpreted together with validation design, dataset representativeness, subgroup analysis, calibration/uncertainty reporting, and clinical usability. Accuracy values above 90% are treated cautiously when they come from internal splits, small or curated datasets, class-balanced benchmarks, augmentation-heavy pipelines, or settings without external validation.

Several studies achieved high diagnostic performance while incorporating explainability. Hasan et al. (5) reported 99.77% accuracy on CT images and 95.37% accuracy on X-ray images for lung abnormality classification using CTXNET and Grad-CAM. Rajpoot et al. (4) reported 99.00% accuracy on chest X-ray images and 96.18% accuracy on CT images for COVID-19 diagnosis while applying LIME, SHAP, Grad-CAM, and Grad-CAM++. Fatima et al. (17), Zou and Miao (16), and Kakon et al. (13) reported high performance in breast cancer and brain tumor studies. These values are useful but should be interpreted in the context of internal validation, dataset curation, and limited external clinical testing.

Responsible AI performance should therefore be judged through multiple criteria: diagnostic discrimination, calibration, subgroup fairness, privacy risk, uncertainty estimation, interpretability validity, external validation, clinician-centered evaluation, workflow integration, and post-deployment monitoring.

## 4 Advancements and evidence distribution across responsible-AI domains

Responsible AI in medical imaging has moved from simple disease classification toward multi-dimensional evaluation. Nevertheless, the included evidence is uneven. Explainability dominates the reviewed studies, whereas fairness, privacy, uncertainty, and clinical-trust studies remain comparatively sparse. This imbalance is now explicitly stated to align the manuscript with the title and abstract.

### 4.1 Explainability: useful but insufficient for clinical trust

Grad-CAM and Grad-CAM++ generate class-discriminative heatmaps and were frequently used because they are easy to visualize. LIME and SHAP provide local perturbation or attribution-based explanations, while saliency maps and RELPROP provide additional visual attribution. However, these tools can be unstable, sensitive to preprocessing, and may highlight regions that correlate with labels without representing clinically valid disease evidence. Therefore, XAI outputs should be treated as decision-support information rather than proof that the model is safe or clinically trustworthy.

Human-centered evaluation is essential. Ihongbe et al. (3) showed that clinicians can perceive XAI positively, but usability depends on explanation clarity, color palettes, cognitive load, potential automation bias, and how explanations fit into radiology workflow. The revised clinical trust section therefore avoids implying that heatmaps alone produce trust.

### 4.2 Fairness and bias analysis

Fairness analysis was expanded to specify which subgroups were assessed in the included studies. Benčević et al. (8) assessed skin tone in skin lesion segmentation and reported a marked reduction in Dice score for dark skin compared with light skin. Lin et al. (2) assessed sex, race, and age in chest radiograph diagnosis and used supervised contrastive learning to reduce demographic bias. Tayebi Arasteh et al. (1) evaluated how privacy-preserving learning affected diagnostic accuracy and fairness. These studies demonstrate that average performance can hide clinically important subgroup disparities.

The review now distinguishes bias measurement from bias mitigation. Measuring bias identifies performance gaps, while mitigation attempts to reduce those gaps through model training, data standardization, subgroup-aware sampling, representation learning, or privacy-fairness optimization. The added self-supervised standardization reference strengthens this point by linking responsible AI to data-standardization strategies for healthcare bias reduction (27).

### 4.3 Privacy-preserving AI

The privacy-preserving AI section was expanded beyond definitions of federated learning and differential privacy. Practical challenges include cross-center data heterogeneity, inconsistent labeling protocols, privacy-utility trade-offs, communication cost, model update governance, membership-inference/model-leakage risks, and the need to document whether privacy-preserving models are externally or clinically validated. Federated learning can reduce the need for direct data sharing, but it does not automatically remove privacy risk. Differential privacy can bound information leakage, but stronger privacy budgets may reduce utility, calibration, or subgroup fairness.

### 4.4 Clinical trust and translation

Clinical trust is a socio-technical outcome, not a single model metric. It depends on radiologist-AI interaction, workflow integration, alert fatigue, the clarity and actionability of model explanations, calibration of confidence estimates, regulatory approval, medico-legal responsibility, post-deployment monitoring, and evidence that AI improves patient outcomes. The revised manuscript therefore adds concrete recommendations for clinical translation rather than assuming that retrospective accuracy and heatmaps are sufficient.

## 5 Methodological quality, risk of bias, and heterogeneity

A structured appraisal was added because the original version did not include a formal quality or risk-of-bias assessment. The appraisal indicates that many included studies have moderate or high concern in at least one domain, especially validation, dataset representativeness, subgroup reporting, calibration, and clinical applicability. This does not invalidate the studies, but it limits the strength of clinical claims.

## 6 External validation, clinical translation, and trust recommendations

External validation was strengthened as a separate clinical translation issue. Most included studies did not provide a clearly independent prospective external validation cohort. Where multi-dataset or subgroup testing was present, performance often varied across domains or patient groups. Because studies used different outcomes and metrics, a pooled performance drop was not calculated.

Clinical translation recommendations. Before responsible AI systems are used broadly in medical imaging, studies should: (1) validate on independent hospitals, scanners, and geographic populations; (2) report subgroup performance by age, sex, race/ethnicity where ethically and legally available, skin tone where relevant, disease severity, scanner type, and hospital site; (3) report calibration, uncertainty, and clinically meaningful thresholds; (4) evaluate explanation quality with clinicians; (5) document privacy-preserving governance and leakage-risk testing; (6) assess workflow integration, alert fatigue, and radiologist-AI interaction; (7) define medico-legal responsibility and regulatory status; and (8) monitor drift and real-world errors after deployment.

## 7 Limitations and future work

This review has limitations. First, it is a PRISMA-informed scoping mini review rather than a full systematic review or meta-analysis, and therefore it maps representative evidence rather than estimating pooled diagnostic accuracy. Second, the search was restricted to studies published between 2020 and 2025 and may not capture all relevant proceedings from IEEE TMI, IEEE JBHI, MICCAI, AAAI, and other venues. Third, the included studies are heterogeneous in disease area, imaging modality, architecture, dataset size, and validation design. Fourth, many performance metrics were not directly comparable across studies. Fifth, because most studies lacked prospective or external validation, the review cannot conclude that responsible-AI tools are ready for broad clinical deployment.

Future research should prioritize multi-center prospective validation, patient-level data splitting and leakage checks, subgroup fairness reporting, privacy threat modeling, uncertainty calibration, clinician-centered XAI evaluation, workflow integration studies, regulatory documentation, and post-deployment monitoring. Future reviews should use a fully registered protocol, duplicate independent screening, publicly available extraction forms, and standardized quality appraisal tools such as QUADAS-2, PROBAST-AI, CLAIM, and TRIPOD-AI/PROBAST-AI when applicable.

## 8 Conclusion

Responsible AI in medical imaging is not limited to building accurate disease classifiers. It requires integrated evidence for diagnostic performance, explainability, fairness, privacy, uncertainty, calibration, external validation, clinical usability, and governance. The 24 included studies show promising performance across lung disease, cancer, brain tumor, diabetic eye disease, and skin disorder applications. However, high retrospective accuracy and visual explanations should not be interpreted as proof of clinical readiness. Safe translation requires independent external validation, subgroup fairness analysis, privacy-risk evaluation, clinician-centered testing, regulatory and medico-legal planning, and post-deployment monitoring.

## Data Availability

The original contributions presented in the study are included in the article/Supplementary Material, further inquiries can be directed to the corresponding author.

## Supplementary material

Supplementary Table S1 Reproducible database search strings and screening information used for the review.
