# Supplementary material for: Responsible artificial intelligence in medical imaging: a systematic review
Source: Front Digit Health. 2026 Jul 16;8:1884692. doi: 10.3389/fdgth.2026.1884692 (PMC13422431; doi:10.3389/fdgth.2026.1884692)
Supplement: Supplementary Table S1 — Reproducible database search strings and screening information used for the review. [file Table1.pdf]

Table S1. Reproducible search strings and source-specific search strategy

Database	Exact search strategy / reproducibility note
PubMed	(("responsible AI"[Title/Abstract] OR "explainable AI"[Title/Abstract] OR fairness[Title/Abstract] OR privacy[Title/Abstract] OR "federated learning"[Title/Abstract] OR "differential privacy"[Title/Abstract] OR uncertainty[Title/Abstract]) AND ("medical imaging"[Title/Abstract] OR radiology[Title/Abstract] OR MRI[Title/Abstract] OR CT[Title/Abstract] OR "X-ray"[Title/Abstract] OR mammography[Title/Abstract] OR ultrasound[Title/Abstract] OR dermoscopy[Title/Abstract] OR "retinal imaging"[Title/Abstract] OR OCT[Title/Abstract]) AND ("disease detection"[Title/Abstract] OR diagnosis[Title/Abstract] OR classification[Title/Abstract] OR segmentation[Title/Abstract])) AND ("2020/01/01"[Date - Publication] : "2025/12/31"[Date - Publication])
Scopus	TITLE-ABS-KEY(("responsible AI" OR "explainable AI" OR fairness OR privacy OR "federated learning" OR "differential privacy" OR uncertainty) AND ("medical imaging" OR radiology OR MRI OR CT OR "X-ray" OR mammography OR ultrasound OR dermoscopy OR "retinal imaging" OR OCT) AND ("disease detection" OR diagnosis OR classification OR segmentation)) AND PUBYEAR > 2019 AND PUBYEAR < 2026

Web of Science	TS=(("responsible AI" OR "explainable AI" OR fairness OR privacy OR "federated learning" OR "differential privacy" OR uncertainty) AND ("medical imaging" OR radiology OR MRI OR CT OR "X-ray" OR mammography OR ultrasound OR dermoscopy OR "retinal imaging" OR OCT) AND ("disease detection" OR diagnosis OR classification OR segmentation)) AND PY=(2020-2025)
IEEE Xplore	("All Metadata":"responsible AI" OR "All Metadata":"explainable AI" OR "All Metadata":fairness OR "All Metadata":privacy OR "All Metadata":"federated learning" OR "All Metadata":"differential privacy" OR "All Metadata":uncertainty) AND ("All Metadata":"medical imaging" OR "All Metadata":radiology OR "All Metadata":MRI OR "All Metadata":CT OR "All Metadata":"X-ray" OR "All Metadata":mammography OR "All Metadata":ultrasound OR "All Metadata":dermoscopy OR "All Metadata":"retinal imaging" OR "All Metadata":OCT) AND ("All Metadata":"disease detection" OR "All Metadata":diagnosis OR "All Metadata":classification OR "All Metadata":segmentation); Years: 2020-2025
ScienceDirect	("responsible AI" OR "explainable AI" OR fairness OR privacy OR "federated learning" OR "differential privacy" OR uncertainty) AND ("medical imaging" OR radiology OR MRI OR CT OR "X-ray" OR mammography OR ultrasound OR dermoscopy OR "retinal imaging" OR OCT) AND ("disease detection" OR diagnosis OR classification OR

	segmentation); Years: 2020-2025; Research articles and reviews
SpringerLink	("responsible AI" OR "explainable AI" OR fairness OR privacy OR "federated learning" OR "differential privacy" OR uncertainty) AND ("medical imaging" OR MRI OR CT OR "X-ray" OR mammography OR ultrasound OR dermoscopy OR OCT) AND ("disease detection" OR diagnosis OR classification OR segmentation); Years: 2020-2025
Google Scholar	allintitle/keyword search combinations of: "responsible AI" OR "explainable AI" AND "medical imaging" AND disease detection; additional targeted searches for fairness, privacy, federated learning, differential privacy, uncertainty, radiology, MRI, CT, X-ray, mammography, dermoscopy, retinal imaging, and OCT; first 100 results screened by relevance
